# Tracking through Life Stages: Adult, Immature and Juvenile Autumn Migration in a Long-Lived Seabird

**DOI:** 10.1371/journal.pone.0072713

**Published:** 2013-08-16

**Authors:** Clara Péron, David Grémillet

**Affiliations:** 1 Centre d’Ecologie Fonctionnelle et Evolutive, CEFE CNRS UMR 5175, Montpellier, France; 2 Percy FitzPatrick Institute, DST/NRF Centre of Excellence, University of Cape Town, Rondebosch, South Africa; Universidad Nacional Autonoma de Mexico, Mexico

## Abstract

Seasonal long-distance migration is likely to be experienced in a contrasted manner by juvenile, immature and adult birds, leading to variations in migratory routes, timing and behaviour. We provide the first analysis of late summer movements and autumn migration in these three life stages, which were tracked concurrently using satellite tags, geolocators or GPS recorders in a long-ranging migratory seabird, the Scopoli’s shearwater (formerly named Cory’s shearwater, *Calonectris* *diomedea* ) breeding on two French Mediterranean islands. During the late breeding season, immatures foraged around their colony like breeding adults, but they were the only group showing potential prospecting movements around non-natal colonies. Global migration routes were broadly comparable between the two populations and the three life stages, with all individuals heading towards the Atlantic Ocean through the strait of Gibraltar and travelling along the West African coast, up to 8000 km from their colony. However, detailed comparison of timing, trajectory and oceanographic conditions experienced by the birds revealed remarkable age-related differences. Compared to adults and immatures, juveniles made a longer stop-over in the Balearic Sea (10 days vs 4 days in average), showed lower synchrony in crossing the Gibraltar strait, had more sinuous pathways and covered longer daily distances (240 km.d^-1^ vs 170 km.d^-1^). Analysis of oceanographic habitats along migratory routes revealed funnelling selection of habitat towards coastal and more productive waters with increasing age. Younger birds may have reduced navigational ability and learn progressively fine-scale migration routes towards the more profitable travelling and wintering areas. Our study demonstrates the importance of tracking long-lived species through the stages, to better understand migratory behavior and assess differential exposure to at-sea threats. Shared distribution between life stages and populations make Scopoli’s shearwaters particularly vulnerable to extreme mortality events in autumn and winter. Such knowledge is key for the conservation of critical marine habitats.

## Introduction

Autumn migration from high latitudes is triggered by decreasing temperatures, food shortage and day length shortening. In moving towards lower latitudes, animals generally track more productive systems and meet more clement conditions, improving their winter survival [1], a key parameter driving population dynamic in long-lived species [2]. However, the migration period is critical since energy demand is high and harsh weather or poor resource availability at stop-over sites can compromise survival of the different demographic components of a population [2,3,4]. Since the advent of miniaturized long-lasting electronic tracking devices, many studies have explored the migratory pathways of both terrestrial [5,6,7] and marine species [8,9,10,11], revealing extraordinary long-distance trans-equatorial migrations [12,13,14]. However, most of these studies have been conducted on adults for methodological reasons [15].

More recently, technological advances have enabled to focus attention on more cryptic life stages, also called the ‘lost years’ [16], including juvenile and immaturity life stages, when animals do not breed and spend most of their time away from breeding grounds for several years [11,15,17,18,19,20,21,22]. Studying movements of individuals belonging to early life stages using electronic devices is yet challenging because they are difficult to access before their first breeding attempt and often experience higher mortality than adults [15,23,24,25]. Moreover, in some species, young life stages are smaller and tend to grow rapidly (e.g. sea turtles), requiring flexible attachment methods to accommodate growth [15]. Being more sensitive to environmental changes, early life stages can thus mitigate or accentuate the effects of extreme environmental events on population dynamic [26]. Tracking animals through life stages has therefore been identified as an important step towards understanding dispersal and behavioural plasticity across all life stages, and develop conservation strategies at the population level [15,22,27]. Conservation measures are currently essentially based on adult distribution during the breeding and/or inter-breeding season [28], although spatial distribution of the other life stages can differ significantly [18,29].

Autumn migration is experienced in a different manner across life stages: juveniles undertake their very first winter journey, whereas adults and immatures usually move towards previous wintering areas [1]. It has been demonstrated that bird migratory behaviour has an inherited genetic component, migration control being primarily based on an endogenous clock-and-compass system during the first outward journey of juveniles [1]. During this first journey, juveniles migrate on their own, learn cues and acquire skills that are used as complementary mechanisms for the control of the spring return journey and further journeys during their adult life [1].

Differential navigational and/or foraging skills combined with intra-specific competition for food resources have been identified as significant components in the evolution of niche width for different life stages [30,31] and can result in spatial segregation. For example, juvenile and adult honey buzzards ( *Pernis* *apivorus* ) shared the same wintering areas [17], whereas the autumn/winter distributions of juvenile wandering albatrosses ( *Diomedea* *exulans* ) [18] and immature common guillemots ( *Uria* *aalge* ) [29] showed little overlap with those of adults. Immatures northern gannets ( *Morus* *bassanus* ) have been found to briefly prospect non-natal colonies and to cover larger distances than breeders during the chick-guarding period [32]. Further, lesser black-backed gulls ( *Larus* *fuscus* ) were geographically segregated by age throughout the entire annual cycle, with immature gulls travelling farther than adults [33,34]. In raptors, telemetric studies revealed that juveniles can migrate along partly different routes [17,35]: they make more or longer stops to replenish their energy stores, take longer to complete their journeys, display more diverse departure directions and more sinuous paths than adults [17,35,36,37]. Differences between life stages have also been documented in timing of migration, with juvenile and immature raptors tending to migrate earlier in the autumn than breeding adults [1,17,36]. Additionally, ontogenic shifts have also been reported in sea turtle diet [19] and habitat choice [11].

However, no study has provided a complete overview of autumn migratory behaviour across adults, immatures and juveniles concurrently in a wild population. In addition, very few studies investigated the movements of immatures during the late breeding season, when they potentially visit natal or non-natal colonies before migrating to winter areas [32]. Such evidence of prospecting movements has important conservation implications because they condition dispersal and recruitment, favouring gene flow and long term population persistence [38].

Procellariiforms contain some of the most globally threatened species of birds in the world, facing multiple threats on land and at sea [39]. Among them, the Scopoli’s shearwater ( *Calonectris* *diomedea* [40]) is one of the four seabird species endemic to the Mediterranean Sea. This species is highly philopatric, has a low reproductive rate and delayed maturity [25]. This later life history trait results in a long immature period, when birds are likely to improve travelling and foraging skills before their first breeding attempts [41]. Scopoli’s shearwaters are classified as *Least Concern* according to the IUCN Red List [42], however they are still facing multiple threats on land (hunting, predation by rats and cats, habitat degradation) and at-sea (bycatch, habitat degradation, competition with fisheries).

Using biotelemetry data collected on Scopoli’s shearwaters from two breeding colonies in the Western Mediterranean Sea, we investigated whether (1) movements of immature birds differ from those of breeders during the late breeding period, (2) autumn migration routes and timing vary between juveniles, immatures and adults and (3) oceanographic preferences along the migration pathways evolve with age/experience.

To our knowledge, this study is the first to investigate differences through life stages in autumn migration of a long distant migrant using biotelemetry data collected simultaneously on three different life stages (adult, immature and juvenile). Although of cross-sectional design, our results provide essential population-level information which clarifies the spatial ecology and the conservation biogeography of a long-lived seabird.

## Material & Methods

### Ethics Statement

Access to protected areas and tag deployments on Scopoli’s shearwaters were approved by the boards of the ‘Conservatoire d’Espaces Naturels de Provence-Alpes-Côte d’Azur’, the ‘Réserve Naturelle de l’archipel de Riou’, the ‘Parc Maritime des îles du Frioul’ and the ‘Réserve Naturelle des Bouches de Bonifacio’. Bird instrumentation was performed under personal animal experimentation permits 34-369 (D. Grémillet) and # A34-505 (C. Péron) delivered by the French ‘Direction Départementale de la Protection des Populations’. Details on data collection are given in the ‘Tracking data’ section.

### Study sites and species biology

**Figure 1:**
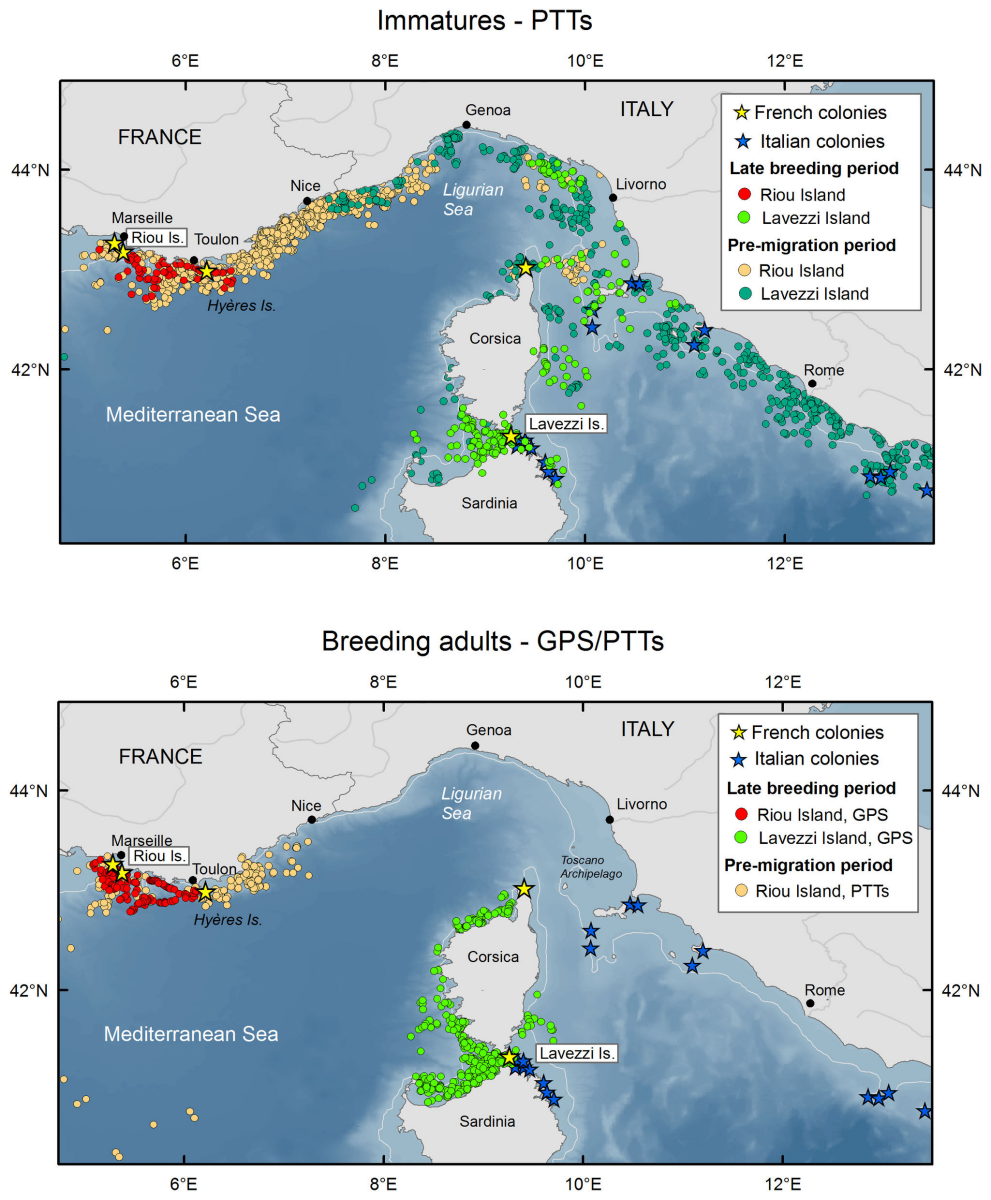
Late-summer and pre-migration distribution of breeding adult and immature Scopoli’s shearwaters. At-sea foraging trips of adults and immatures tracked during the late breeding season (20/08/2011–12/09/2011) and pre-migration period (12/09/2011 onwards).

Our study was conducted in 2011-2012 on three French Mediterranean islands hosting breeding colonies of Scopoli’s shearwaters: the Marseille islands (Riou and Frioul) in Provence and Lavezzi Island in south Corsica (Figure 1). Riou and Lavezzi islands host the largest French colonies for this species, with approximately 300 and 350 breeding pairs, respectively, whereas Frioul Island hosts approximately 70 pairs [43]. Long-term demographic monitoring programs on Scopoli’s shearwaters have been conducted on these three islands since 1978 by local wildlife managers. The oldest bird ringed as an adult is currently 35 years old. Scopoli’s arrive at breeding sites in late February and start incubating a single egg in early June, for 52 days on average. Chicks hatch in mid-July and fledge in early October. The autumn migration of breeding adults starts after chick fledging, in mid-October. There is high population-level synchrony in egg-laying, as well as for chick-fledging. This species is highly philopatric [44]; Immatures visit their natal colonies for several years before attempting to breed, supposedly acquiring experience and local knowledge while assessing the merits of potential nesting sites [25,45]. On Lavezzi Island, most immatures return to the colony at five and six years old, but the maximum probability of first breeding was reached at the age of seven [25]. Ages of first return and first breeding are similar for Scopoli’s breeding on Riou Is. (C. Péron, unpublished data). Here, we specifically define immatures as individuals younger than 7 years old that were never observed breeding.

Like Cory’s, Scopoli’s shearwaters forage for fish, squid and crustaceans in the upper meters of the pelagic domain [46] but they can also feed on offals when associated to fishing vessels [47].

### Tracking data

Our study was based on tracking data collected using GPS recorders, geolocators [48] and satellite transmitters (Platform Terminal Transmitters, PTTs), to yield the most complete picture of shearwater migration ecology. Indeed, GPS recorders are most useful to accurately record movements but those used in this study had limited battery power and were therefore only deployed on adults for single foraging trips towards the end of the breeding season. Secondly, geolocators are extremely powerful tools to track year-round movements, but geographical positions have low resolution and cannot be estimated during the equinox period (March and October), when day length is constant throughout the globe [49]. These periods correspond to shearwater autumn and spring migration, and therefore to gain information about these critical phases we equipped birds from the three different life stages with solar-powered PTTs. These tags are nonetheless far more expensive than geolocators and GPS recorders, which constraints mass-deployments across life stages of a population.

Between August and October 2011, we deployed 24 PTTs (Microwave Telemetry, Columbia, USA) simultaneously on 12 juveniles, nine immatures and three breeding adult Scopoli’s shearwaters from Riou, Frioul and Lavezzi islands (Table 1). Juveniles were equipped just before fledging in early October at both Riou (nine individuals) and Frioul (three individuals) islands (Table 1). Adults were equipped at Riou Is. in early October whereas, immatures were equipped from mid-August to mid-September 2011 at Riou and Lavezzi islands (Table 1). When captured on colonies, the identity of immature birds was checked in the demographic databases. Immature birds were equipped if they were banded as chick, younger than 7 years old and never observed as breeders in the colony. These conditions could be filled for six individuals whereas two others were not banded. They were considered as immatures from behavioural cues recorded at night by experts on the colonies (repeated visits, entering empty burrows, vocalization, displaying, absence of brood patch).

**Table 1:** Details of the 21 PTT deployments on Scopoli’s shearwaters in late summer 2011.

ID No.	Status	Site	Age (years)	Body mass (g)	Tag weight (g)	Date of first Argos fix	Date of last Argos fix	Max. range (km)	Total distance (km)	Total duration (days)	Number and percentage of locations kept after filtering	No. resampled locations
73586	Adult	Riou	>10*	650	18	05/10/2011	22/11/2011	4515.6	9631.7	48.3	361 (74.8%)	1163
74440	Adult	Riou	>10*	640	18	06/10/2011	14/10/2011	304.7	1220.1	8.4	86 (72.5%)	204
74485	Adult	Riou	>10*	600	18	05/10/2011	09/01/2012	3740.0	9338.9	96.5	718 (86.4%)	2321
73590	Immature	Lavezzi	4	610	18	18/08/2011	21/09/2011	251.1	2288.7	33.1	117 (57.1%)	797
74351	Immature	Lavezzi	4	625	18	07/09/2011	04/12/2011	5322.2	12894.0	87.8	585 (84.7%)	2112
74447	Immature	Lavezzi	nb	-	18	07/09/2011	31/10/2011	394.1	3682.2	54.1	336 (66.6%)	1302
74450	Immature	Lavezzi	5	-	18	17/08/2011	27/09/2011	341.8	2318.7	41.4	211 (83.2%)	996
73583	Immature	Riou	nb	550	18	07/09/2011	22/11/2011	3834.2	8405.4	76.3	531 (79.0%)	1835
73589	Immature	Riou	5	670	18	09/09/2011	11/11/2011	3549.2	7436.6	63.1	449 (75.8%)	1518
74441	Immature	Riou	6	585	18	09/09/2011	08/12/2011	5431.5	11471.8	89.8	584 (81.1%)	2159
74476	Immature	Riou	6	570	18	10/09/2011	10/11/2011	3878.8	6547.9	61.1	500 (84.7%)	1471
74442	Juvenile	Frioul	0	655**	18	12/10/2011	18/11/2011	4350.5	6830.3	36.6	297 (85.8%)	880
74484	Juvenile	Frioul	0	850**	18	08/10/2011	20/10/2011	595.8	941.4	11.4	164 (89.3%)	274
73582	Juvenile	Riou	0	750	18	08/10/2011	12/12/2011	4815.1	12544.2	65.8	531 (88.0%)	1582
74443	Juvenile	Riou	0	860	18	09/10/2011	19/11/2011	4821.6	8266.9	41.6	346 (88.1%)	1002
74465	Juvenile	Riou	0	730	18	06/10/2011	14/10/2011	28.8	230.3	8.4	75 (78.1%)	204
74467	Juvenile	Riou	0	830	18	07/10/2011	16/10/2011	906.3	1174.6	8.6	132 (87.7%)	207
74486	Juvenile	Riou	0	780	18	14/10/2011	13/11/2011	4043.2	5961.3	29.9	226 (83.9%)	719
74487	Juvenile	Riou	0	700	9.5	11/10/2011	11/11/2011	2781.6	4323.5	31.2	135 (74.3%)	752
74489	Juvenile	Riou	0	715	9.5	11/10/2011	30/10/2011	3464.1	3965.4	19.3	67 (80.8%)	465
74509	Juvenile	Riou	0	760	9.5	16/10/2011	03/11/2011	562.7	1441.6	17.4	91 (67.2%)	420

* indicates three adults ringed as adult in 2007, 2004 and 2008, respectively. All were breeding with a chick in 2008 and considered to be at least seven years old (average age of sexual maturity) leading to a minimum estimated age > 10 in 2011. Immatures were banded as chick except 2 individuals (nb). ** weight measured on the 26^th^ of September 2011, not just before deployment.

PTTs weighed 18g (2.6% of body mass; 3.1% when associated with a geolocator), except three PTTs deployed on juveniles that weighed 9.5g (1.3% of body mass). They were attached to back feathers using Tesa^©^ tape and Loctite^©^ glue. They were powered by solar panels which induced a duty cycle of 10h ON/48h OFF for the 9.5g-PTT and 12h ON/24h OFF for the 18g-PTT.

We also tracked 25 breeding adults from Riou and Lavezzi islands during the late chick rearing period (16^th^ August -11^th^ September 2011) using GPS loggers (Perthold Engineering LLC, weighing 20g) to compare their at-sea movements with those of immatures equipped with PTTs at the same time. Since GPS sampling frequency (2-3 minutes) was higher than the transmission frequency of PTTs, we resampled GPS locations to one position per hour.

On Riou Island, 41 adults were equipped with geolocators (LOTEK Wireless, LAT2500, Canada) during the breeding season 2011 to define their 2011-2012 wintering areas. Among them, three were also fitted with the aforementioned PTTs in October, at the end of the chick-rearing period. These geolocators weighed 3.6g (0.5% of body mass) and were mounted on a darvic ring.

### PTT track reconstruction

We first used a speed-filtering procedure (sda filter [50]) to discard erroneous Argos locations. The maximum mean velocity was set to13 m.sec^-1^, a threshold calculated from high-resolution GPS tracking data from Scopoli’s shearwater (C. Péron, unpublished data). This filtering procedure resulted in 20% of discarded locations in average (Table 1). We then applied a state-space framework to model animal movements while admitting errors in the observation of bird location, and to reconstruct tracks during the OFF period of PTT transmission schedule (24h or 48h). The process model describing animal movement was the first-difference correlated random walk model described by Jonsen et al. [51]. A Bayesian statistical framework was used to estimate probability distributions for the states (animal locations) and movement parameters. The model was fitted to each individual bird with a one-hour time step for tracks reconstruction. Analyses were performed using the free software R [52] and JAGS using the package ‘bsam 0.42’ [51]. Wide, flat, prior probability distributions were assumed for model parameters. A total of 80,000 Monte Carlo Markov-Chains iterations were recovered from two chains (40,000 iterations per chain). The first 30,000 iterations of each chain were discarded as a burn-in, while one every 10^th^ value of the remaining 10,000 iterations was retained to reduce autocorrelation. The final sample size on which inferences were drawn was thus 2,000 iterations. We checked convergence of the error parameter estimates using the potential scale reduction factor,$\hat{R}$; values close to one were consistent with convergence [53]. $\hat{R}$ was <1.01 for all parameter estimates.

### Spatial analyses

Geolocators fitted and recovered on adults provided one location per day with an estimated error of ~180 km [54]. We selected locations corresponding to the winter period (1^st^ November 2011 -1^st^ March 2012) and filtered equinox and erroneous locations using a speed filter [50] that discarded in average 35% of the locations. We then calculated kernel density contours with a smoothing parameter of 1° to estimate the intensity of utilization in their winter quarters.

**Figure 2:**
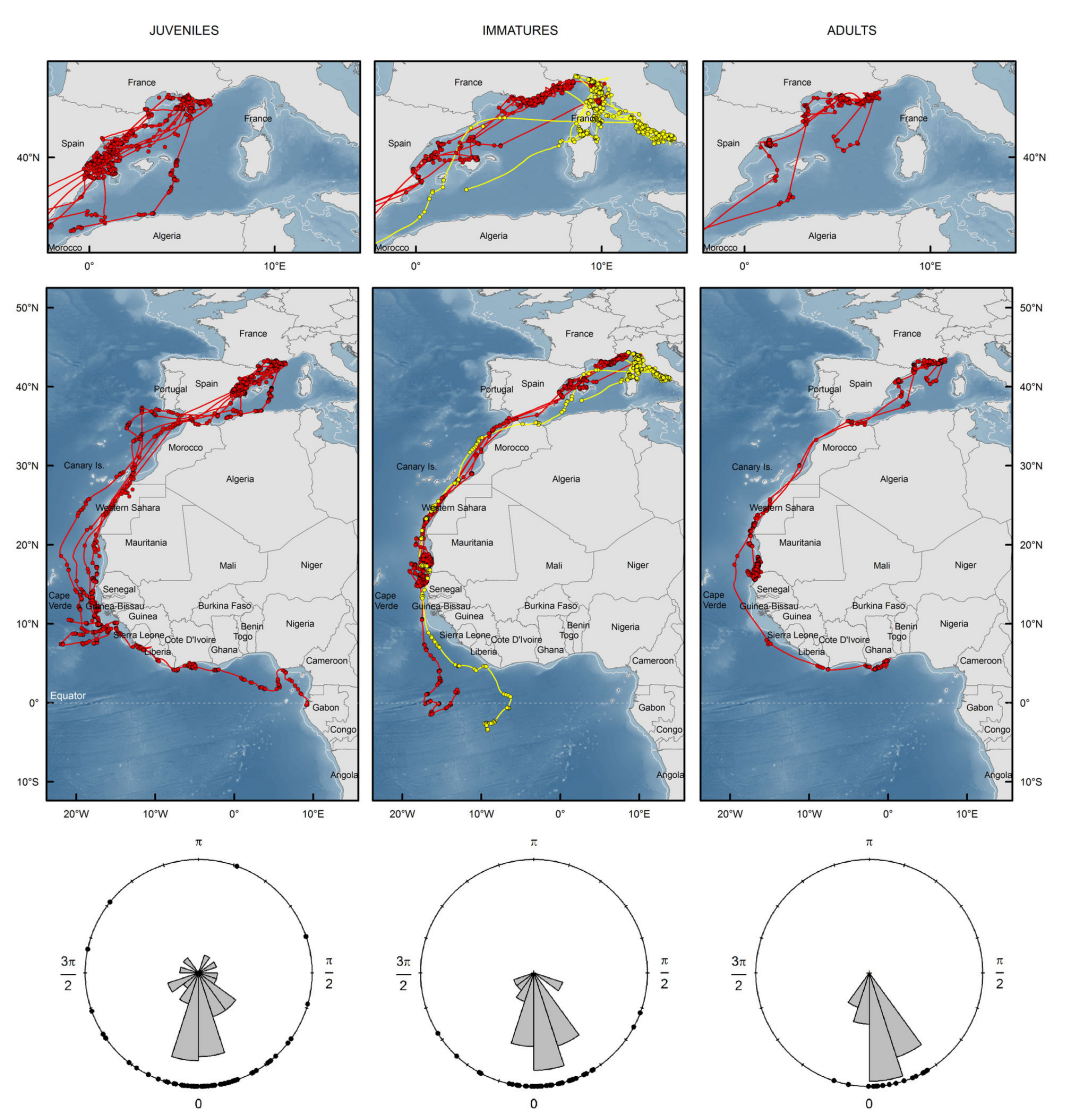
Comparison of juvenile (n=10), immature (n=7) and post-breeding adult (n=3) Scopoli’s shearwaters migratory routes. Upper panel: start of autumn migration/post-fledging departure in the Mediterranean Sea. Middle panel: entire autumn migration/post-fledging departure and arrival at wintering areas. Birds were fitted with PTTs on the Marseille islands (red) and Lavezzi Island, Corsica (yellow). Locations are Argos locations filtered with a speed filter and paths are ‘reconstructed’ tracks using a state-space model. Bathymetry is shown as a blue scale. Lower panel: rose diagrams of turning angles distribution drawn for each life stage highlight inter-stage differences in sinuosity of migratory routes.

For PTT tracks, each location of the ‘reconstructed’ tracks was assigned to a geographical zone to compare timing of migration and synchrony in geographical distribution between individuals of the same group and between groups (juveniles, immatures and adults). The estimated date of passage through the strait of Gibraltar (36.14° N, 5.35° W) was determined by visual inspection of the reconstructed tracks. Life stage comparisons of daily distance travelled were tested using analysis of variance (ANOVA) on log transformed data to meet normality. Sinuosity of migratory pathways after crossing Gibraltar was estimated for each life stage using rose diagram of turning angles calculated between two successive locations, when speed was > 10 km.h^-1^, corresponding to migratory movements. Straightness of the migratory path was assessed by calculating turning angles distributions when travelling (Figure 2). We tested for life stage differences in mean direction and dispersion of turning angles using Rao’s test for homogeneity of angular data [55].

Life stage specific habitat use was compared by plotting two-dimensional kernel density estimation of bathymetry and monthly chlorophyll-a concentration extracted along reconstructed trajectories. Bathymetry was downloaded from the ETOPO2v2 database at a spatial resolution of 0.033° and chlorophyll-a concentration (Aqua MODIS NPP 0.05°, mg.m^-3^) was obtained from BloomWatch website (http://coastwatch.pfel.noaa.gov/coastwatch/CWBrowserWW360.jsp). Both environmental datasets were interpolated on a regular 0.1°×0.1° grid before extraction of environmental variables along the trajectories.

## Results

**Figure 3:**
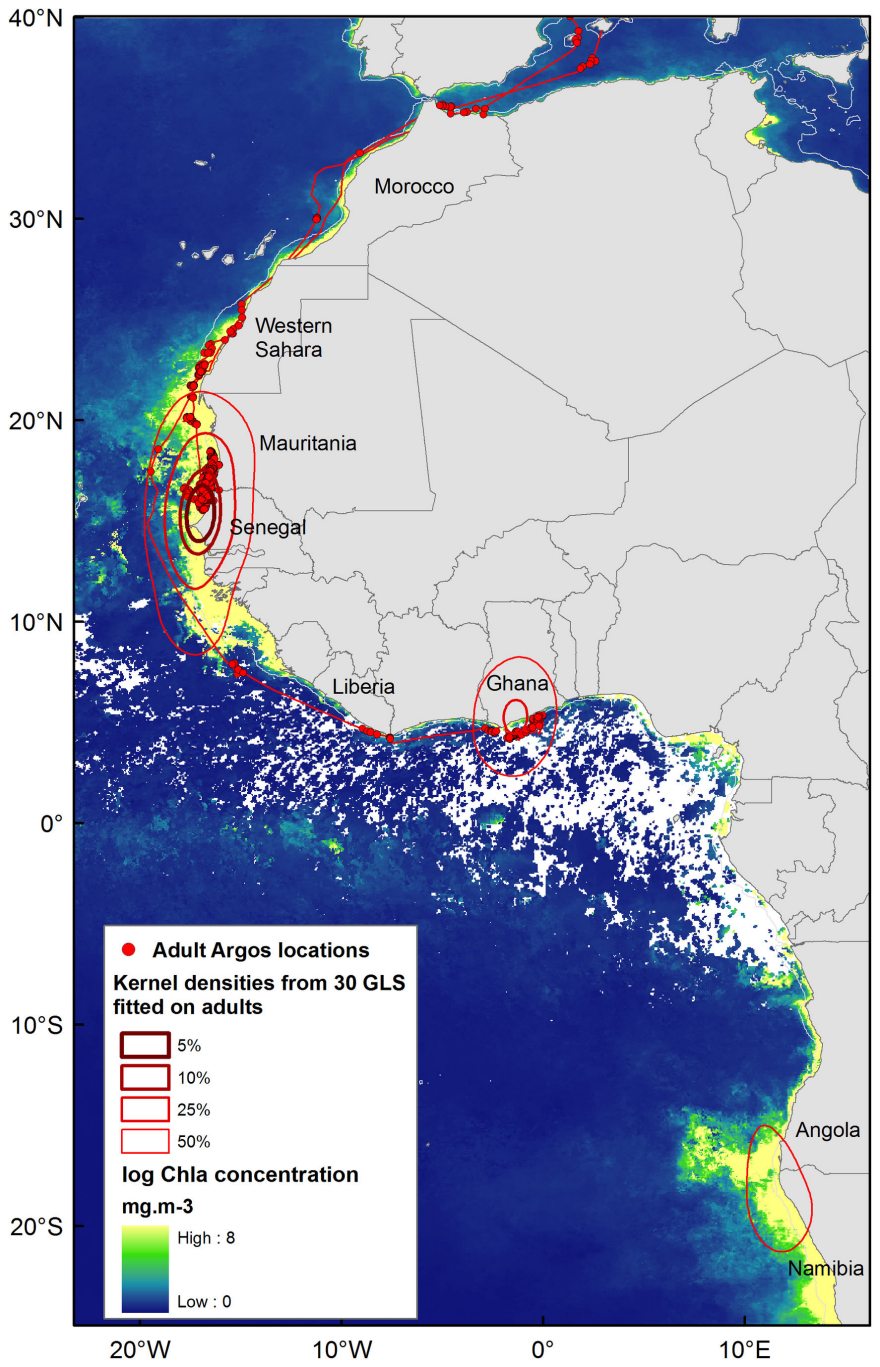
Winter distribution of post-breeding adult Scopoli’s shearwaters. Kernel density contours illustrate the three main wintering areas of 30 adults fitted with geolocators. Argos PTT locations and reconstructed path appear in red dots and lines. The average primary productivity (Chlorophyll-a concentration, mg.m^-3^) for the’r’r winter period (November 2011 to February 2012) is displayed in the background.

A total of ten juveniles, eight immatures and three adults were tracked simultaneously using PTTs. Three PTTs fitted to two juveniles and one immature bird failed to transmit locations. Transmission failures can result from logger detachment, death of the bird or device failure. Total transmission duration was highly variable between equipped birds, ranging from eight days to three months (Table 1). Additionally, seven GPS tracks were obtained from four adults breeding at Riou Is., as well as 34 GPS tracks from 25 adults breeding at Lavezzi Is. (see Table S1 for details). These tracks were recorded during the late breeding season, when immatures were also being tracked using PTTs. Thirty-four out of the 41 geolocators deployed in 2011 were recovered in 2012 on adult Scopoli’s from Riou Island. Among them, 30 were downloaded properly, providing winter locations of adults (Figure 3), including those of two adults equipped with both PTT and geolocator (Figure S1).

### Pre-migration movements of breeders and immatures

Both immatures and breeders from Lavezzi and Riou islands performed short trips over the continental shelf during the late breeding period (20^th^ August -20^th^ September 2011). Off Riou Is., breeding adults and immatures exploited the same foraging zones, south and east of Marseille, whereas the foraging areas of immatures from Lavezzi Is. differed partially from those of adults (Figure 1). Adults from Lavezzi Island foraged mainly in the Bonifacio strait, Gulf of Asinara (Sardinia) or along the west coast of Corsica, whereas the four immature birds stayed mainly in the strait of Bonifacio close to the colony, or travelled to the Italian or Sardinian coasts (Figure 1). After the 20^th^ of September, breeding birds were no longer tracked by GPS, and PTTs indicated that immatures left the Lavezzi Island and foraged along the Italian coast, from Roma to the Liguro-Provencal current (Figure 1). Immature Scopoli’s shearwaters from Riou Island were also present in the Liguro-Provencal current at that time, yet spatial analyses showed that tracks from both groups seldom overlapped. Breeding adults equipped with PTTs at Riou Island in early October also travelled east towards Nice before heading west to Spanish waters (Figures 1 and 2).

Interestingly, only immature birds visited or passed nearby non-natal colonies, i.e. at least once within a buffer of 5 km around non-natal Scopoli’s shearwater colonies. All immatures from Lavezzi Island remained at least once around colonies located in north Corsica, off Sardinia (Maddalena, Tavolara archipelago), in the Tuscany archipelago or other islands along the Italian coast (Figure S2). The four immatures from Riou Island also went within 5 km from the Scopoli’s colonies of the Hyères Islands. Most of the locations within this radius occurred at night (53.4% and 74% for Lavezzi and Riou islands, respectively, Figure S2).

### Autumn migration and wintering areas across the three life stages

**Figure 4:**
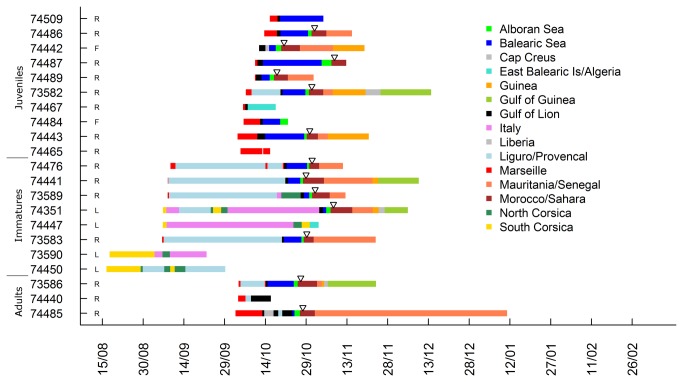
Timing of post-fledging movements of juvenile and autumn migration of immature and adult Scopoli’s shearwaters tracked with PTTs. Triangles correspond to the date of crossing of the Gibraltar strait and the capital letter indicate the colony where birds were equipped (R: Riou Is., F: Frioul Is. and L: Lavezzi Is.).

In early October, we tracked simultaneously migration movements of juvenile birds during their post-fledging journey and those of immatures and adults released from the constraint of central place foraging. At a large spatial scale, Scopoli’s shearwaters of the three life stages headed southwest, crossed the strait of Gibraltar and subsequently travelled south along the West African coastline (Figure 2). Autumn migration therefore consisted in two phases: the first weeks spent in the Mediterranean Sea, followed by rapid directional movements towards wintering areas in the Atlantic Ocean (Figures 2 and 3). In the Western Mediterranean Sea, most juveniles from Marseille islands travelled straight towards the Balearic sea/Iberian shelf waters where they stopped for a period of 10 days in average (range: 2-22 days) (Figures 2 and 4). They exploited waters of the Ebro delta, the gulf of Valencia, Cape La Nao and waters around Eivissa Island (Figure 2). Only one individual headed south to the Algerian coast without crossing the Balearic Sea. Another juvenile first travelled east of the Marseille islands as adults and immatures did, before heading to the Balearic Sea.

Immatures from Riou Island also stopped in the Balearic Sea but for a shorter period (4 days in average, range: 1-7 days) and one bird travelled along the northern coast of Mallorca Island. Breeding adults from Riou Island travelled to Spanish waters in the gulf of Rosas and Ebro delta (Balearic Sea) where one bird stayed for ten days (Figures 2 and 4).

Six juveniles, five immatures and two adults were still equipped with PTTs after crossing the strait of Gibraltar. Juveniles travelled south to Senegal and Guinean waters. One juvenile went to the gulf of Guinea and transmission stopped 1.5 months after fledging (on 12^th^ of December) when it crossed the equator off Gabon (Figure 2).

The five immatures stopped in waters off Mauritania and Senegal, where they spent several weeks. Two of them continued their migration southwards in offshore equatorial waters (Figure 2). Among adults, PTT and geolocator data highlighted the importance of coastal waters located off Mauritania and Senegal during winter (Figures 2 & 3). One bird equipped with PTT stayed between Cap Vert (Dakar, Senegal) and Nouakchott, Mauritania for more than two months (transmissions from the 1^st^ November to the 12^th^ of January). Geolocator devices revealed two other wintering areas exploited by thirteen adults (43%): the gulf of Guinea and the northern Benguela current (off Angola and Namibia). One adult tracked with PTT flew directly towards a restricted area in coastal waters off Ghana, a wintering area shared by four others adult Scopoli’s tracked with geolocators (Figures 2, 3 and S1). Interestingly, most birds of the three life stages travelled and/or foraged in a narrow corridor within 20 km from the coast of Western Sahara (Figure 2).

### Inter-stage differences in migratory timing and behavior

The date of passage through the strait of Gibraltar was more variable for juveniles than for other groups (31^th^ October 2011 on average). The first Scopoli’s shearwater passing through Gibraltar Strait on the 18^th^ of October 2011 was a juvenile and the last ones crossing the strait on the 8^th^ of November 2011 were one juvenile and one immature (Figure 4). Crossings of immatures and adults through Gibraltar were highly synchronized in late October (28^th^ of October 2011 in average) compared to juveniles (Figure 4).

Stop-overs were more frequent and lasted longer in juveniles than in adults and immatures (Figure 4). Juveniles spent up to three weeks in the Balearic Sea, whereas immatures and adults stopped for one to ten days (Figure 4).

**Figure 5:**
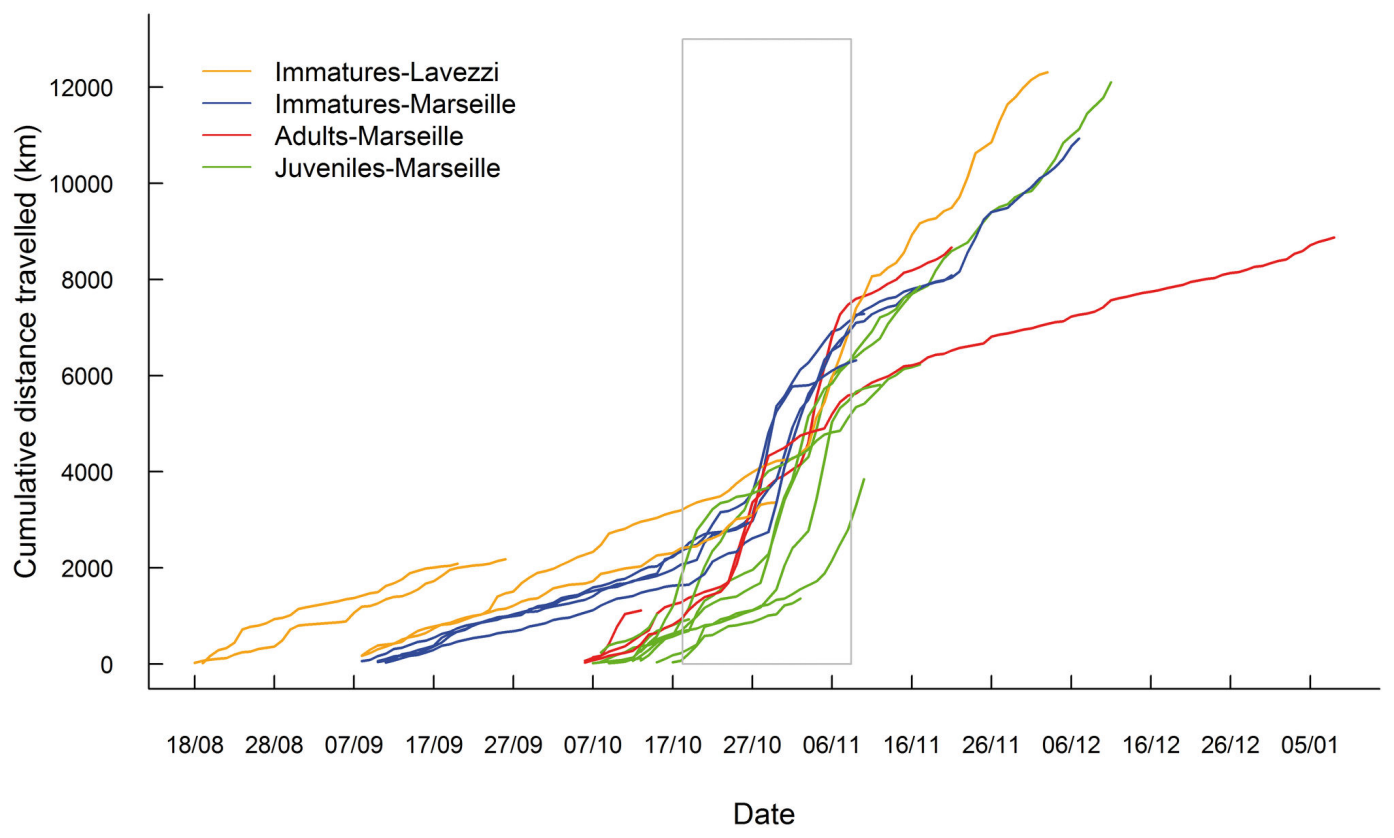
Comparison of cumulative travelled distances between life stages. Daily distance was calculated from individual PTT tracks reconstructed using state-space models for juvenile, immature and adult Scopoli’s shearwaters. The rectangle corresponds to the period when birds crossed the strait of Gibraltar.

Daily travelled distance was significantly higher in the Atlantic (199.8 km.day^-1^) than in the Mediterranean Sea (90.5 km.day^-1^, F_1,874_=150.4, P<0.001). Juveniles travelled at the same pace as adults and immatures soon after fledging (Figure 5). However, when travelling in the Atlantic Ocean, daily travel distance was significantly higher for juveniles (239.6 ±180 km.d^-1^, n=6 individuals) compared to adults and immatures (177.4 ±182 km.day^-1^, F_1,874_=46.09, P<0.001, n=7 individuals). Rao’s test revealed that there was no difference in the general direction of migration route through the stages (T_2_=2.55, P>0.1), but the dispersion parameter (variance of the turning angles) was significantly higher for juveniles compared to adults and immatures (T_1_=11.23, P<0.001 and T_1_=5.54, P<0.05, respectively). Adults and immatures had similar dispersion parameters of turning angles (T_1_=3.24, P=0.07).

### Inter-stage differences in habitat use during migration

**Figure 6:**
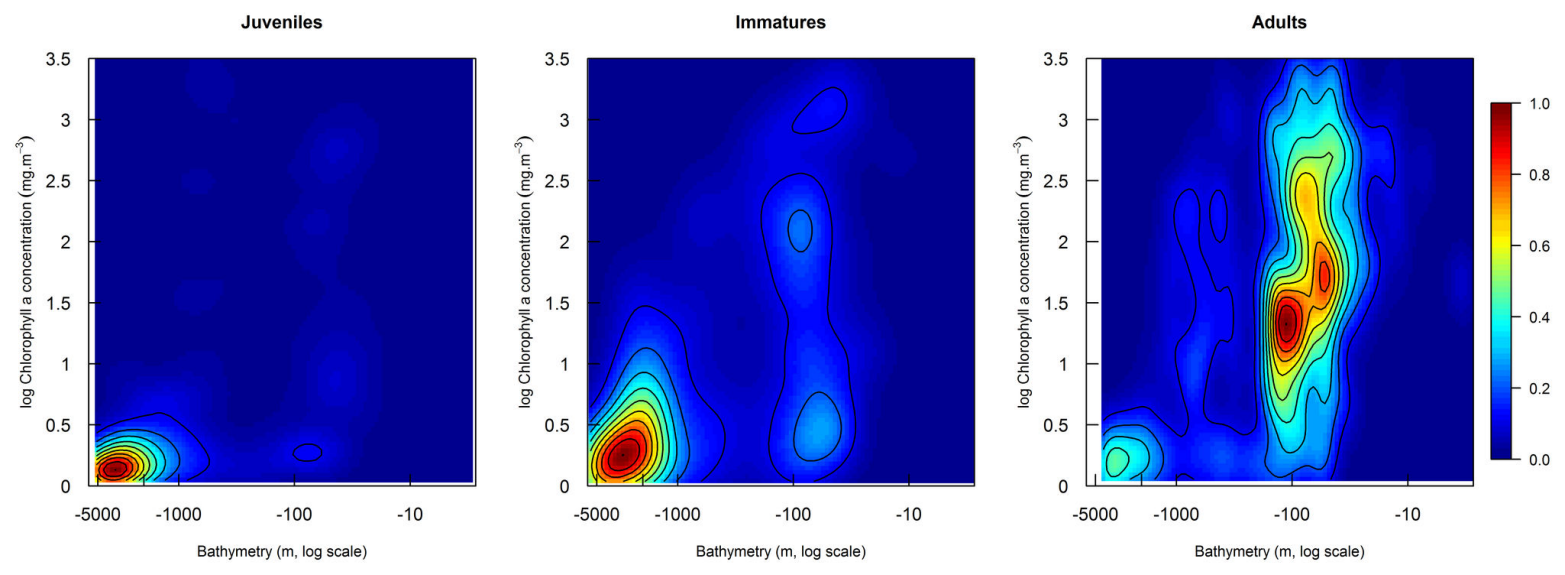
Comparison of migratory oceanographic preferences between life stages. Two-dimensional niches were plotted from kernel density estimations of bathymetry and chlorophyll-a concentration extracted along reconstructed trajectories of Scopoli’s shearwaters migrating from Gibraltar to winter quarters along the Western African coast. The color scale corresponds to scaled density estimation (d-min(d)/(max(d)-min(d)) for each individual life stage.

Two-dimensional kernel density estimation of habitat use highlighted life stage differences in oceanographic conditions experienced by juveniles, immatures and adults while travelling in the Atlantic Ocean (Figure 6). Adults travelled in coastal waters along the continental shelf (<100 m depth) where chlorophyll-a concentration was higher, whereas juveniles, and to a lesser extent immatures, travelled over deeper, less productive waters (Figure 6).

## Discussion

By using an array of biotelemetry technologies simultaneously on three different life stages, we had the rare opportunity to gain essential knowledge on age-related differences in migratory behavior of Scopoli’s shearwaters. We found similar at-sea movements between immatures and breeders during the late breeding season, except that immatures were more prone to prospecting movements nearby non-natal colonies. Juvenile, immature and adult birds did differ in their autumn migratory paths, timing and behavior and the different age groups studied showed different oceanographic preferences along their migration paths. One study group (PTT-tracked adults) was based on a limited sample size (three adults in the Mediterranean, two in the Atlantic), yet, importantly, their highly consistent coastal travelling behaviour is strongly supported by previous PTT tracking of adult Scopoli’s shearwaters [56] and their equally highly coastal, winter destinations confirmed by geolocator data from 30 individuals (Figure 3).

### Immature and breeding adult movements during the late summer

Our results show that immature birds caught at their natal colony foraged close by during summer, similarly to breeding adults. Accuracy and data transmission rate of the Argos system prevent estimation of the frequency of nighttime visits to the colony but short foraging radii and repeated sightings on the colony suggest that immatures behaved like adults (Figure 1). However, immature Scopoli’s shearwaters left the vicinity of the colony before chick fledging, exploring more distant waters along the Liguro-Provencal current or the Italian coast. In contrast with shared wintering areas, immature Scopoli’s shearwaters from the two different colonies showed a clear spatial segregation in October, before migration onset (Figure 1).

Interestingly, most immatures flew at least once nearby non-natal breeding colonies at night, suggesting prospecting behaviour and potential for dispersal. Although the actual dispersal rate of Scopoli’s shearwater among colonies is poorly known and likely to be very low across Mediterranean colonies [44], evidence of regular prospecting movements in immature birds is an important finding, since minimum gene flow promoted by dispersal is sufficient to connect distinct populations [38]. In agreement with Gómez-Díaz et al. [57], it suggests that the Mediterranean population of shearwaters may function as a meta-population. This result has far reaching conservation implications, especially in the context of breeding habitat degradation caused by introduced predators in most colonies of Scopoli’s shearwaters [58].

### Autumn migration

The Balearic Sea appears as an important stop-over site for juvenile Scopoli’s shearwaters in October. The Iberian shelf waters of the Balearic Sea are also exploited by adult Scopoli’s shearwaters breeding on Balearic Islands in July–August and by the *Critically Endangered* balearic shearwaters ( *Puffinus* *mauretanicus* ) in May–June [59,60]. The Balearic Sea, particularly the Ebro Delta area, is a highly productive region with major pelagic fish spawning areas [61,62]. Juveniles may also feed on fishery discards in this region of high fishing activity [63,64].

When reaching the Atlantic Ocean, Scopoli’s shearwaters from the three life stages initiated oriented movements along the West African coast, towards their wintering areas. Off Mauritania, Senegal, and in the gulf of Guinea, movement paths were shorter and path sinuosity higher in all three life stages compared to movements performed during the commuting phase along the Atlantic coast. Such behavioural changes suggested a resident phase when birds forage in productive areas and rest on the water in their wintering areas (Figure 3). Mauritania continental shelf is a very productive upwelling region [65], known as wintering ground for many seabird species [66,67]. Studies using geolocators fitted to adult Cory’s and Scopoli’s shearwaters from different populations in the Atlantic Ocean and Mediterranean Sea previously highlighted the importance of the Canary current, Mauritania and Senegal coastal waters as wintering areas [13,68]. After a stop-over of two to three weeks in this area, two immatures started an exploring phase in deep equatorial waters, while one juvenile explored the coast of the gulf of Guinea. In contrast, adults were resident to specific foraging areas (Mauritania, Senegal, Ghana or Angola/Namibia) for the entire winter period (November–February) as suggested by Argos PTT locations and confirmed by geolocator data (Figures 2, 3 and S1).

### Stage-specific migratory behaviour and habitat preferences

Adult and immature Scopoli’s shearwaters showed higher synchrony than juveniles in October migration (Gibraltar crossing), suggesting a tighter response of the two former groups to environmental cues (e.g. wind and temperature fields). After fledging, juveniles spent substantially more time in the nearby Balearic Sea. This finding is in agreement with previous raptor migration studies, which showed that juveniles make longer stops to replenish their energy stores, potentially because of lower foraging efficiencies [17,36,37].

Interestingly, juveniles were able to fly long daily distances soon after fledging (Figure 5), in contrast with juvenile wandering albatross which progressively increased their daily flight distances and attained adult flight efficiency within their first six months at sea [18]. When travelling in the Atlantic Ocean, juvenile Scopoli’s shearwaters covered longer average daily distance than immatures and adults.

Higher turning angles dispersion in juveniles, compared to immatures and adults, when travelling to their winter areas attested that juveniles followed a more sinuous day-to-day route, as in two species of raptors [17,37]. In birds, juveniles are assumed to perform initial movements away from the native colony using innate navigational skills with a fixed flight direction and time program [1,69]. Imperfections in this innate migratory behaviour might explain why juvenile Scopoli’s shearwaters showed less developed navigational skills compared to adults and immatures, particularly when entering the Atlantic Ocean. The susceptibility of juvenile birds migrating solitarily to extensive wind drift has already been demonstrated in Thorup et al. [70], and probably leads to increased mortality during the first year at sea. Navigational abilities of young birds, especially their capacity to compensate for wind drift, might be less developed than in mature adults, therefore leading to long flyways, higher energy expenditure and potentially lower survival probability. Indeed, Jenouvrier et al. [25] found an annual survival rate of 0.52 during the first year compared to 0.88 and 0.89 for immature and adult Scopoli’s shearwaters from Lavezzi Island, respectively.

Differences in the use of bathymetry and chlorophyll-a concentration between the three life stages suggest a progressive funnelling of migratory pathways, leading more experienced individuals to use more direct pathways along the coastline of narrow continental shelf of West Africa, where ocean productivity is higher (Figure 6). Adult Scopoli’s shearwaters tracked by Argos PTTs from a Greek colony [56] showed similar, highly directional and coastal migratory paths, which support our findings. This inter-stage difference in oceanographic habitat use (Figure 6) suggests either learning of navigational skills during the first post-fledging years and/or selection of individuals targeting shallower waters where productivity is higher. Further studies are needed to understand what cues are used during the first migration of juvenile shearwaters, and how their navigation system develops during the first years at sea. Birds may use visual, magnetic, and/or olfactory cues to refine their habitat towards coastal and productive waters [1]. For instance, it has been shown that petrels and shearwaters use dimethyl sulphide (DMS), a volatile sulfur compound emitted by phytoplankton as a source of information to find rich food patches at upwelling zones over continental shelf areas (e.g. [71]).

### Conservation implications

At a large spatial scale, low inter-individual and inter-stage differences in autumn migration routes and wintering areas of Scopoli’s shearwaters suggest that different populations and the three demographic components of the populations experience similar environmental conditions and potential anthropogenic threats during these periods. This finding has strong conservation implications because synchronization of mortality across population subsets (age classes, sex) is likely to decrease population growth rate and increase extinction risk in long-lived species whose population growth is mainly driven by adult survival [2,72]. In this context, Jenouvrier et al. [73] found high synchronization of inter-annual adult survival rate across five populations of Scopoli’s shearwaters from the Mediterranean Sea. These authors suggested that the different populations were exposed to similar environmental conditions during the non-breeding period, and that climatic events such as La Niña years, when Atlantic hurricanes and storms are stronger, could be responsible for synchronized increases in adult mortality [73]. Our results support this hypothesis since we demonstrated that individuals from two populations across all life stages would be affected if extreme environmental conditions, major bycatch events in fisheries, or a major oil spill would occur in autumn in the Gibraltar Strait, Balearic Sea or off the West African coast. This migratory strategy prevents compensatory recruitment [29], a mechanism documented in common guillemots. In this species, the larger home-range of young individuals enabled populations to buffer the impact of major oil spills that occurred within restricted adult home-range, through higher recruitment of immatures after selective adult mortality [29]. As marine pollution and extreme meteorological events show marked spatial variations, such spatial segregation between stages decrease extinction risk at the population level.

Similarly, the three life stages of Scopoli’s shearwaters probably face the same exposure to fisheries bycatch, in contrast to other procellariiform species which showed spatial segregation between stages [18,22]. The Balearic Sea and the Ebro delta support one of the most important fishing fleets of the Western Mediterranean Sea and Scopoli’s shearwaters are frequently caught accidentally [74,75]. A previous study reported that most of the Scopoli’s shearwaters caught by longliners in the Balearic Sea were between 4 and 13 years old in summer [74]. However, juvenile Scopoli’s shearwaters are also likely to be accidental victims of fisheries when foraging in this area in October during their first weeks at sea. Further threats from fisheries probably occur all along the migration route of the three life stages off West Africa: the Canary and Guinea currents are very productive zones attracting extensive pelagic fisheries [76] and the coast of Mauritania is one of the richest fishing zones in the world [65]. Moreover, the current political situation in Western Sahara is preventing this new nation from managing its marine resources, and numerous foreign vessels are currently operating in its national waters, without any control over fishing quotas and bycatch of non-target organisms (http://www.wsrw.org). There are major concerns that substantial numbers of migrating shearwaters are being caught by these illegal fisheries [77], especially inexperienced juveniles.

Finally, climate change is a growing threat to the persistence of long-lived seabirds [78]. An increase in the frequency of extreme climatic events is already perceptible [79] and adverse consequences of climate change may threaten species which, like the Scopoli’s shearwater, have little scope for compensatory recruitment [29].

In this context, our study highlights the importance of considering the movements and distribution across age classes of a population to fully understand factors affecting population dynamics and adjust conservation actions to the spatio-temporal variations of the distribution of long-lived migratory animals.

## Supporting Information

Table S1 Summary of GPS tracks of adult breeder Scopoli’s shearwaters tracked in the late chick rearing period in Riou Is. and Lavezzi Is.
(DOC)

Figure S1 **Comparison of Argos PTT tracks and locations estimated from geolocators in 2 adult Scopoli’s shearwaters tracked simultaneously with the two devices.** Red and green locations correspond to Argos fixes filtered with a speed filter and paths ‘reconstructed’ using a state-space model. PTT transmitted from the 5^th^ October to the 22th of November 2011 for the individual in red and from the 5^th^ of October to the 9^th^ of January for the individual in green. The geolocators were fitted to the bird the 10^th^ of August 2011 (individual in red) and the 3^th^ of October 2011 (individual in green). Both geolocators were recovered the 27^th^ of March 2012. Geolocators confirmed the resident behaviour of adults during the non-breeding period. Locations on continents are erroneous, due to the low accuracy of geolocation (~180 km). Bathymetry is shown as a blue scale.
(TIF)

Figure S2 **Percentage of Argos PTT locations of immature Scopoli’s shearwaters found within an increasing radius around non-natal colonies during nightime.** The numbers appearing on the graph correspond to the number of individuals of each colony (black: immatures from Riou Is. visiting Hyères Is. colonies and red: immatures from Lavezzi Is. visiting Italian or other Corsica colonies).
(TIF)
