# Supplementary material for: Tracking through Life Stages: Adult, Immature and Juvenile Autumn Migration in a Long-Lived Seabird
Source: PLoS One. 2013 Aug 16;8(8):e72713. doi: 10.1371/journal.pone.0072713 (PMC3745401; doi:10.1371/journal.pone.0072713)
Supplement: Table S1 — Summary of GPS tracks of adult breeder Scopoli’s shearwaters tracked in the late chick rearing period in Riou Is. and Lavezzi Is. (DOC) [file pone.0072713.s001.doc]

Table S1: Summary of GPS tracks of adult breeder Scopoli’s shearwaters tracked in the late chick rearing period in Riou Is. and Lavezzi Is.

| **Bird id** | **GPS trip id** | **Site** | **start date (GMT)** | **return date (GMT)** |
| --- | --- | --- | --- | --- |
| ID10L | 1 | Lavezzi | 03/09/2011 02:39 | 05/09/2011 18:27 |
| ID11L | 1 | Lavezzi | 08/09/2011 02:57 | 09/09/2011 02:17 |
| ID12L | 2 | Lavezzi | 07/09/2011 03:21 | 07/09/2011 20:34 |
| ID13L | 1 | Lavezzi | 07/09/2011 03:31 | 09/09/2011 01:12 |
| ID16L | 1 | Lavezzi | 16/08/2011 22:01 | 20/08/2011 18:02 |
| ID20L | 1 | Lavezzi | 16/08/2011 22:01 | 24/08/2011 18:53 |
| ID21L | 1 | Lavezzi | 31/08/2011 03:39 | 03/09/2011 03:16 |
| ID22L | 1 | Lavezzi | 07/09/2011 23:11 | 08/09/2011 23:56 |
| ID22L | 2 | Lavezzi | 09/09/2011 03:51 | 11/09/2011 01:41 |
| ID26L | 1 | Lavezzi | 17/08/2011 01:50 | 18/08/2011 18:30 |
| ID29L | 1 | Lavezzi | 17/08/2011 21:39 | 19/08/2011 19:09 |
| ID29L | 2 | Lavezzi | 19/08/2011 20:08 | 20/08/2011 17:52 |
| ID30L | 1 | Lavezzi | 27/08/2011 21:49 | 28/08/2011 20:25 |
| ID30L | 2 | Lavezzi | 28/08/2011 21:17 | 29/08/2011 19:14 |
| ID30L | 3 | Lavezzi | 29/08/2011 22:33 | 30/08/2011 18:42 |
| ID31L | 1 | Lavezzi | 05/09/2011 22:27 | 07/09/2011 01:40 |
| ID35L | 1 | Lavezzi | 06/09/2011 03:56 | 06/09/2011 22:45 |
| ID38L | 1 | Lavezzi | 20/08/2011 01:36 | 20/08/2011 18:26 |
| ID38L | 2 | Lavezzi | 21/08/2011 02:21 | 21/08/2011 19:17 |
| ID42L | 1 | Lavezzi | 21/08/2011 03:29 | 21/08/2011 18:11 |
| ID42L | 2 | Lavezzi | 22/08/2011 03:13 | 22/08/2011 22:06 |
| ID4L | 1 | Lavezzi | 16/08/2011 22:01 | 17/08/2011 00:03 |
| ID4L | 2 | Lavezzi | 17/08/2011 00:57 | 19/08/2011 01:14 |
| ID4L | 3 | Lavezzi | 19/08/2011 01:38 | 19/08/2011 20:25 |
| ID5L | 1 | Lavezzi | 22/08/2011 02:46 | 22/08/2011 18:30 |
| ID5L | 2 | Lavezzi | 23/08/2011 01:24 | 24/08/2011 18:57 |
| ID6L | 1 | Lavezzi | 28/08/2011 02:24 | 28/08/2011 19:46 |
| ID6L | 2 | Lavezzi | 29/08/2011 02:46 | 29/08/2011 18:34 |
| ID6L | 3 | Lavezzi | 30/08/2011 03:20 | 30/08/2011 19:14 |
| ID7L | 1 | Lavezzi | 03/09/2011 23:12 | 04/09/2011 15:07 |
| ID8L | 2 | Lavezzi | 16/08/2011 22:02 | 17/08/2011 19:56 |
| ID9L | 1 | Lavezzi | 26/08/2011 02:33 | 26/08/2011 20:39 |
| ID9L | 2 | Lavezzi | 27/08/2011 02:58 | 27/08/2011 19:43 |
| ID9L | 3 | Lavezzi | 28/08/2011 03:11 | 28/08/2011 18:14 |
| ID30R | 4 | Riou | 06/09/2011 22:00 | 07/09/2011 00:03 |
| ID3R | 1 | Riou | 07/09/2011 03:14 | 09/09/2011 01:27 |
| ID3R | 2 | Riou | 09/09/2011 02:08 | 10/09/2011 02:24 |
| ID5R | 1 | Riou | 07/09/2011 00:56 | 08/09/2011 20:11 |
| ID7R | 1 | Riou | 07/09/2011 03:31 | 07/09/2011 22:01 |
| ID7R | 2 | Riou | 08/09/2011 03:52 | 08/09/2011 21:25 |
| ID7R | 3 | Riou | 09/09/2011 03:42 | 10/09/2011 01:50 |
