# Supplementary figures and images for: Tracking through Life Stages: Adult, Immature and Juvenile Autumn Migration in a Long-Lived Seabird
Source: PLoS One. 2013 Aug 16;8(8):e72713. doi: 10.1371/journal.pone.0072713 (PMC3745401; doi:10.1371/journal.pone.0072713)

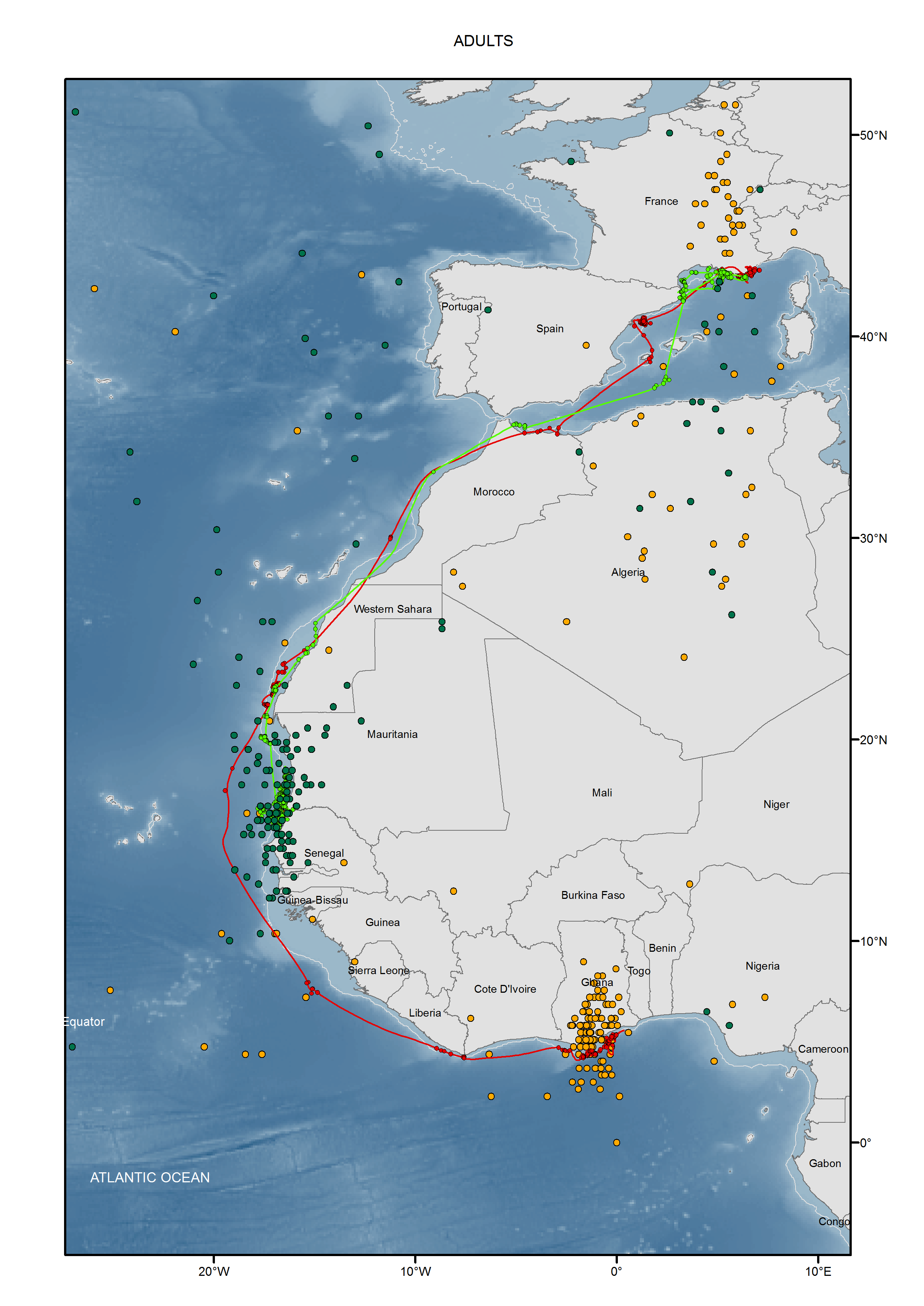

Supplement: Figure S1 — Comparison of Argos PTT tracks and locations estimated from geolocators in 2 adult Scopoli’s shearwaters tracked simultaneously with the two devices. Red and green locations correspond to Argos fixes filtered with a speed filter and paths ‘reconstructed’ using a state-space model. PTT transmitted from the 5th October to the 22th of November 2011 for the individual in red and from the 5th of October to the 9th of January for the individual in green. The geolocators were fitted to the bird the 10th of August 2011 (individual in red) and the 3th of October 2011 (individual in green). Both geolocators were recovered the 27th of March 2012. Geolocators confirmed the resident behaviour of adults during the non-breeding period. Locations on continents are erroneous, due to the low accuracy of geolocation (~180 km). Bathymetry is shown as a blue scale. (TIF) [file pone.0072713.s002.tif]
